# Validity and reliability study of the Turkish version of the Hand20 questionnaire

**DOI:** 10.3906/sag-1808-168

**Published:** 2019-08-08

**Authors:** Nurten Gizem TORE, Deran OSKAY

**Affiliations:** 1 Department of Physiotherapy and Rehabilitation, Faculty of Health Sciences, Gazi University, Ankara Turkey

**Keywords:** Upper extremity injuries, hand, patient reported outcome measures.

## Abstract

**Background/aim:**

This study aimed to translate and validate the Turkish version of the Hand20 questionnaire.

**Materials and methods:**

Patients who had upper extremity involvement and stable symptoms for the previous 4 weeks in their upper extremities were included in the study. Patients who were illiterate or used a splint during the day were excluded from the study. Participants completed the Turkish version of the Disabilities of the Arm, Shoulder, and Hand (DASH-T) questionnaire once and the final version of the Hand20 questionnaire twice in a 7-day interval. Internal consistency and reliability of the questionnaire was assessed. Moreover, correlations between Hand20 and DASH-T scores were analyzed using Spearman’s correlation coefficient.

**Results:**

A total of 104 patients participated in the study. The Turkish version of the Hand20 met the set criteria of reliability and validity. Internal consistency (Cronbach’s alpha = 0.93) and test-retest reliability were excellent (r = 0.82). Hand20 showed a positive and statistically significant correlation with DASH-T (r = 0.76, P < 0.001).

**Conclusion:**

The results showed that the Turkish version of the Hand20 had excellent test-retest reliability and validity. As a result of this study, it was determined that Hand20 was a valid and reliable instrument to measure the upper extremity disabilities of Turkish-speaking patients.

## 1. Introduction

Outcome measurements, which have great importance in evaluating the results of treatment, can be divided into 2 groups; clinician-focused tests and patient-based outcome measures. Clinician-focused tests have been used to assess physical functions, such as range of motion, muscle strength, and sensory functions, in upper extremity disorders. In these tests, objective results have been reflected, but subjective results, such as a patient’s perceptions, pain, or daily living activities, have not been [1,2]. Therefore, patient-based outcome measurements were developed to evaluate the level of a patient’s disability and allow for comprehensive evaluation.

Numerous patient-based outcome measurements for the upper extremities have been developed. Some were generalized for the upper extremities, while others were joint-specific [3] or disease-specific [4]. However, deciding the most compatible instrument to assess the affected upper extremity is difficult [5].

One reason for this difficulty is that most of the instruments, such as questionnaires and scales, were developed in English-speaking countries [2]. However, in order to obtain the validity of translated instruments, a merely linguistic translation is inadequate. Cultural adaptation is also required. Hence, some specific methodologies have been developed for the cross-cultural adaptation process [6]. By virtue of these methodologies, the effect of a disease or a treatment could be explained in a similar way in different countries [6,7].

Hand20 was developed by orthopedic surgeons at the Department of Hand Surgery of Nagoya University’s School of Medicine to assess upper extremity disorders. During the process of item selection, Suzuki et al. tried to choose items that appeared to reflect the function of the upper extremities and were the least affected by cultural differences. It is composed of 20 short questions accompanied by explanatory illustrations that elderly people and children can easily understand (19 of the 20 questions) [8]. Although many questionnaires exist that assess disorders of the upper extremities, to our knowledge, no questionnaires evaluate upper extremity disorders in individuals both younger than 18 years and older than 65 years. Hand20 can be easily applied to individuals under 18 years old and over 65 years old, thanks to the fact that, as mentioned earlier, it has short and easy-to-understand questions that are accompanied by explanatory illustrations [8]. The features of Hand20 that are highlighted above distinguish it from other upper extremity questionnaires. Hence, the translation and the cross-cultural adaptation of Hand20 have important roles. 

The aim of this study was to perform a cross-cultural adaptation of the Hand20 questionnaire and investigate whether it is a valid and reliable instrument for assessing the outcomes of upper extremity disorders among Turkish-speaking people.

## 2. Materials and methods 

### 2.1. Translation procedure

Before starting the study, permission was received from the author who had developed the original version of the Hand20 questionnaire. The translation and cross-cultural adaptation of Hand20 were done according to the guidelines provided by Beaton et al. [6]. The original version of the questionnaire was translated into Turkish by 2 native Turkish speakers, one of whom was a physiotherapist informed about the study while the other was an English linguistics expert uninformed about the study. The translators were fully competent in both English and Turkish. Translators turned the 2 Turkish translations into a single translation. The Turkish version of the translation was translated back into English by 2 independent professional bilingual translators. A committee, which consisted of 4 translators, a Turkish linguist, and a methodologist, made some slight changes for cultural adaptation because some words had different meanings in Turkish. The comprehensibility of Hand20 was tested on 20 patients with an upper extremity disorder and 20 healthy individuals after the committee agreed that the English and Turkish versions of Hand20 were equivalent. Participants reported that they could understand all of the questions well and the final Turkish version of Hand20 was created. 

### 2.2. Patients

The study included 104 patients (48 females, 56 males; mean age: 43.18 ± 16.71 years; range: 15 to 80 years) with a variety of upper extremity disorders. Patients were referred to the outpatient Department of Physiotherapy and Rehabilitation at Gazi University, Faculty of Health Sciences. The study was conducted between November 2016 and May 2017. The inclusion criteria were: 1) being diagnosed with any upper extremity pathology, 2) being literate in Turkish, 3) being 15 years of age or older, 4) having stable symptoms for the previous 4 weeks, and 5) receiving no treatment between the test-retest assessments. The exclusion criteria were: 1) having bilateral disorders of the upper extremity and 2) using a splint during the day. Signed informed consent was obtained from all of the participants. Signed informed guardian consent was also obtained for participants under 18 years of age. 

The study was approved by the Ethics Committee of the Gazi University Health Sciences Institute. All of the participants filled in the Turkish version of the Disabilities of Arm, Shoulder, and Hand (DASH-T) questionnaire once to assess the validity of Hand20. In order to determine the test-retest reliability, all of the patients completed Hand20 for a second time in a 7-day interval. 

### 2.3. Assessment of reliability and validity

After signing an informed consent form, all of the participants completed the Turkish versions of the Hand20 and DASH questionnaires during their first visit to the outpatient Department of Physiotherapy and Rehabilitation at the Gazi University Faculty of Health Sciences.

DASH is an upper extremity-specific outcome measure that has been shown to be a reliable and valid patient-reported outcome in patient populations with various upper extremity disorders, and it has been translated into several languages [9]. It evaluates impairments, activity limitations, and participation restrictions for leisure activities and work from a patient’s perspective. It comprises 21 items that evaluate difficulties with specific tasks, 5 items that evaluate symptoms, and 1 item that evaluates sleep and confidence, social function, and work function. DASH produces scores between 0 and 100. A high DASH score indicates severe disability [9]. In this study, DASH-T was chosen to assess the validity of Hand20 because it assesses the upper extremities in general, without discriminating according to the regions, and the subparameters of DASH evaluate the limitations of the upper limbs in detail. The Turkish version and cross-cultural adaptation of DASH was performed by Düger et al. [10]. 

Hand20 was developed to assess upper extremity disorders. It comprises 20 short, easy-to-understand questions accompanied by explanatory illustrations (19 of the 20 questions) to boost comprehensibility. Thus, it can be used for patients above 65 and under 18 years of age [8]. Hand20 scores range from 0 to 100, where a high Hand20 score indicates more disability [11]. The questionnaire has been validated in patients with several upper extremity pathologies and injuries [5]. 

### 2.4. Statistical analysis

Statistical analysis of the data was performed using SPSS 22.0 (IBM Corp., Armonk, NY, USA). Statistical data were expressed as the mean ± standard deviation (X ± SD), median, or percent (%). The single-sample Kolmogorov–Smirnov test was used to show the parametric or nonparametric distribution of the data. Test-retest and internal consistency analyses were conducted to determine the reliability of the Hand20 questionnaire. Test-retest reliability was analyzed using Spearman’s *ρ* correlation coefficients. The internal consistency of Hand20 was assessed using Cronbach’s alpha**coefficient. The construct validity of Hand20 was assessed by correlating the total scores with the total scores of DASH-T using Spearman’s *ρ* correlation coefficients. Spearman’s *ρ* correlation coefficients ranging between 0.81 and 1.00 were considered excellent, while 0.61–0.80, 0.41–0.60, 0.21–0.40, and 0–0.20 were accepted as very good, good, weak, and bad, respectively [12]. Statistical significance was accepted as P < 0.05.

## 3. Results

### 3.1. Patient characteristics

As a result of the analysis, it was found that most of the patients were male, unemployed, and high school graduates. It was also determined that the majority of the patients’ dominant hand was the right hand and their affected extremity was also the right. Demographics and clinical characteristics of the patients are presented in Table 1. 

**Table 1:** Demographic features and clinics of the participants.

	n = 104
Age (years)	43.18 ± 16.71
	n (%)
Sex	
Female	48 (46.2)
Male	56 (53.8)
Employment status	
Employed	49 (47.1)
Unemployed	55 (52.9)
Education	
Primary school	14 (13.5)
Middle school	22 (21.2)
High school	50 (48.1)
University	15 (14.4)
Postgraduate	3 (2.9)
Dominant hand	
Left	7 (6.7)
Right	97 (93.3)
Affected limb	
Left	49 (47.1)
Right	55 (52.9)

The participants had various upper extremity complaints, including tendon and nerve injuries, carpal tunnel syndrome, and fractures. The diagnoses of the participants are shown in Table 2.

**Table 2:** Diagnosis of the participants by number and percent.

Diagnosis	n	%
Hand tendon injury	15	14.4
Nerve injury	11	10.6
Carpal tunnel syndrome	11	10.6
Fracture	10	9.6
Both hand tendons and nerves injury	9	8.7
Impingement syndrome	6	5.8
Injuries of muscles, nerves, arteries, and tendons of the hand together	6	5.8
Amputation	5	4.8
Dorsal ganglion	5	4.8
Hand osteoarthritis	4	3.6
Replantation	3	2.9
Tendon injury accompanied with fracture	3	2.9
Brachial plexus injury	3	2.9
Frozen shoulder	3	2.9
Medial epicondylitis	2	1.9
Kienböck syndrome	2	1.9
Dislocation	2	1.9
Transplantation	1	1
Bicipital tendinitis	1	1
Trigger finger	1	1
Lateral epicondylitis	1	1
Total	104	100

### 3.2. Completeness of the item responses 

All of the Hand20 questions were considered clear by most of the participants. Descriptive statistics for the Hand20 and DASH-T scores gained during the first and second interviews are summarized in Table 3. The existing guidelines on the DASH website indicate that if a participant leaves more than 10% of the questions unanswered, it should be defined as inappropriate. On Hand20, up to 2 questions were left blank by 2 participants (1.92%). However, 3 or more questions on DASH-T were left blank by 28 participants (26.92%). As for Hand20, Q8: Roll up and squeeze a towel hard and Q12: Hang wet clothes on a clip hanger were the unanswered questions. For DASH-T, Q4: Preparing a meal, Q8: Garden or outdoor property work, Q18: Recreational activities-tennis, Q19: Recreational activities-tipcat, and Q21: Sexual activities were the most unanswered questions. 

**Table 3:** Median and range of the Hand20 and DASH scores.*

Instrument scale	M (IQR)	Range
Hand20 (1st interview), n = 104	19.25	11–38.37
Hand20 (2nd interview), n = 104	15.25	8–29.87
DASH, n = 104	22.41	12.49–35.27

*Hand20 score, sum of n responses/n × 10; DASH score, [(sum of n responses/n – 1) × 25]; n, number of completed responses.

### 3.3. Reliability and validity 

The internal consistency of Hand20 was assessed using Cronbach’s alpha coefficient. It was high for the 20 items on Hand20 (0.933). When calculating the Cronbach’s alpha coefficient for each of the 20 items by eliminating each item one by one, the range was between 0.92 and 0.93. It was found that none of the items changed the internal consistency. The Cronbach’s alpha coefficients if an item was excluded and the total coefficient of Hand20 are shown in Table 4.

**Table 4:** Alpha coefficients if excluding the item, and total alpha of the dimensions.

1. Wash your face with both hands	0.928
2. Cut all 10 nails on the fingers of both hands properly (using a nail clipper)	0.927
3. Do up shirt buttons with both hands	0.928
4. Pick coins out of a purse with the affected hand	0.928
5. Turn on/off the faucet with the affected hand	0.927
6. Open a milk carton with both hands	0.928
7. Open a plastic bottle	0.931
8. Roll up and squeeze a towel hard	0.929
9. Peel an apple using a knife	0.928
10. Operate a door knob and open a heavy door with the affected hand	0.927
11. Push a heavy object up and onto the shelf overhead using both hands (about 5 kg)	0.930
12. Hang wet clothes on a clip hanger	0.928
13. Wash your hair with both hands	0.927
14. Turn over pages of a newspaper with the affected hand	0.931
15. Do manual work without too much difficulty	0.931
16. Do you hesitate to show people your affected hand for cosmetic reasons?	0.935
17. Do you experience difficulties in recreational activities (painting, knitting, sports)?	0.930
18. Do you experience difficulties in activities of daily living?	0.930
19. How much pain do you have in your affected hand?	0.938
20. Do you feel less confident because of your affected hand?	0.935
Total	0.933

For the test-retest reliability, all of the participants completed Hand20 for the second time with a 7-day interval. No statistically significant differences were found between the test-retest scores of Hand20 (median score (interquartile range): 19.25 (11–38.37) versus 15.25 (8–29.87)). The correlation analyses revealed a Spearman’s rho correlation coefficient of 0.821, P < 0.001. A score that was equal to 1 meant excellent correlation, while one that was equal to 0 meant no correlation [12]. Considering the results of the analyses, the findings indicated that Hand20 had excellent test-retest reliability. 

The correlation between the Hand20 and DASH-T scores was analyzed for construct validity. A statistically significant and positive correlation was found between the Hand20 and DASH-T scores (Spearman’s *ρ* correlation coefficient, 0.761, P < 0.001). This result showed that Hand20 had very good correlation with DASH-T. 

## 4. Discussion

The Turkish translation and cultural adaptation of the Hand20 questionnaire was carried out following a systematic standardized approach. Hand20 is a region-specific, Japanese illustrated questionnaire for disorders of the upper extremities, comprising 20 short, easy-to-understand items accompanied by explanatory illustrations. The validation of Hand20 was proved by Suzuki et al. by comparing Hand20 with the DASH questionnaire [8]. 

In this study, Cronbach’s alpha value for Hand20 was 0.933, demonstrating excellent internal consistency. As far as it is known, only the Greek version of the translated Hand20 has been published, and the authors reported the Cronbach’s alpha coefficient for their version as 0.956 [5]. In the original version, Suzuki et al. reported the Cronbach’s alpha value as 0.973 for the 20 items [8]. Our results for the Cronbach’s alpha coefficient were similar. A Cronbach’s alpha value of greater than 0.70 is considered significant [13]. Excellent internal consistency in multidimensional questionnaires indicates that the questions in different dimensions are strongly correlated.

The test-retest reliability of Hand20 was satisfying with a Spearman’s *ρ *correlation coefficient of 0.821. Goula et al., in their study of the Greek version, also analyzed the test-retest reliability with Spearman’s *ρ *correlation coefficient and reported it as 0.971 [5]. In the original version, Suzuki et al. assessed test-retest reliability using the intraclass correlation coefficient, which in their study was 0.943 [8]. The result of the Spearman’s *ρ *correlation coefficient for the test-retest reliability in the present study was lower than the results of both Goula et al. and Suzuki et al. However, if a result was higher than 0.81, it could be accepted as excellent for correlation analyses [12]. Therefore, the Turkish version of Hand20 demonstrated excellent test-retest reliability, which suggested the stability of Hand20 over time and the consistency of the total scores between the 2 assessments.

The correlation between the Hand20 and DASH-T scores was analyzed to assess the construct validity of Hand20, with Spearman’s *ρ *= 0.761. Goula et al. also analyzed the construct validity with Spearman’s *ρ *correlation coefficient by comparing Hand20 with DASH and reported it as 0.749 [5]. Suzuki et al. found excellent correlation between the Hand20 and DASH questionnaires. The correlation coefficient was 0.91 for their study [8]. The result in the present study was slightly higher than that of Goula et al. and lower than that of Suzuki et al., indicating very good correlation between the Hand20 and DASH questionnaires.

With regards to the completeness of the questions, Hand20 had remarkably better rates of response than DASH. Some questions on both questionnaires were left unanswered by all of the participants due to differences in sex. For Hand20, Q8 and Q12 had the highest unanswered rate among the participants. More men than women left Q8 (12.5% of 56 males) and Q12 (21.4% of 56 males) unanswered because “Roll up and squeeze a towel hard” and “Hang wet clothes on a clip hanger” were considered as household female duties, in compliance with Turkish culture.

Regarding the DASH questionnaire, a detailed analysis indicated that since the participants had not experienced the activities, they did not answer Q8: Garden or outdoor property work, Q18: Recreational activities-tennis, and Q19: Recreational activities-tipcat. For these questions, there was a remarkably higher unanswered rate for female participants. A reason for this discrepancy seen in Q18 and Q19 might be that in this study the mean age of the women was 46.10. In Turkey, most generally, women at around this age are not interested in sports. A reason for Q8 was that it was considered as a male task, according to Turkish culture. Q4: Preparing a meal was one of the most significantly higher unanswered questions among male participants. It was considered as a household female task, in compliance with Turkish culture. Apart from these, Q21: Sexual activities was the most unanswered question among the participants, especially for women (75% of the 48 female participants). 

The existing guidelines on the DASH website point out that it is recommended for evaluation disorders of the upper extremities in patients aged between 18 and 65 years [8]. Our findings demonstrated that patients with a wider range of age (between 15 and 80 years) replied better to the Hand20 questionnaire. Given the increasing elderly population in the world, there is a growing need for tools to assess the health-related quality of life of older people. One of the most momentous advantages of the Hand20 questionnaire is that it can be easily completed by individuals over the age of 65 [5,8]. In our study, similar findings were obtained.

A responsiveness analysis, which is an important measure in determining sensitivity to clinical changes in quality of life questionnaires, was not performed in this study, which was the most consequential limitation of this study. A responsiveness study of Hand20 and validation studies of Hand20 for specific diseases are suggested for future studies.

In conclusion, this study demonstrated that the Hand20 questionnaire is highly valid and reliable as a useful instrument for evaluating a variety of upper extremity disorders seen in Turkish patients. In accordance with other studies assessing this tool, Hand20 was found to be a concise, easy-to-understand, and comprehensible questionnaire with illustrated questions.
